# The Benign Renal Masses that Were Exposed after Nephron-Sparing Surgery: “Postsurgical Fatty Tumor.” Is It Related to the Surgical Technique?

**DOI:** 10.15586/jkcvhl.v9i1.195

**Published:** 2021-11-28

**Authors:** Mehmet Balasar, Mehmet Serkan Özkent, Arif Aydin, Hakan Hakkı Taskapu, Ahmet Atici, Gokhan Ecer, Mehmet Giray Sonmez

**Affiliations:** 1 Department of Urology, Meram Medical Faculty, Necmettin Erbakan University, Konya, Turkey;; 2 Department of Urology, Konya City Hospital, Konya, Turkey

**Keywords:** angiomyolipoma, benign renal neoplasm, nephron-sparing surgery, partial nephrectomy, postsurgical fatty tumor, renal cancer

## Abstract

After nephron-sparing surgery (NSS), postsurgical fatty tumor could be mistakenly reported as angiomyolipoma during radiologic imaging of some patients. In the present paper, we studied the postsurgical fatty tumor detected after NSS but not covered before in the literature. In addition, we also evaluated whether the postsurgical fatty tumor was related to the surgical technique employed. Patients admitted to the urology department of our university hospital from 2014 to 2019 and operated with open NSS were evaluated retrospectively. We detected those 156 patients were operated with NSS. Nine patients with angiomyolipoma as primary pathology and four patients with surgical border positivity were excluded from the study. The patients were divided into two groups based on the repair of tumor extraction region. In Group 1, fatty tissue was used for repair, and Group 2 is the primary repair group. In all, 143 patients (Group 1 = 79, and Group 2 = 64) were included in the study. No demographic and radiologic differences, such as number of patients, age, gender, positioning of tumor, mass localization, tumor diameter, and RENAL nephrometry scoring system, were detected between the two groups. Postsurgical fatty tumors were detected in 28 patients in Group 1 and in two patients in Group 2 (P < 0.001). In patients with negative surgical margins after partial nephrectomy, lesions that were radiologically detected mimicking as angiomyolipoma were defined as “postsurgical fatty tumor.” This mass containing adipose tissue only neither depicted vascularization and enhancement nor increase in size for at least 1 year. We assumed that these lesions must be followed as benign lesions not requiring additional treatment.

## Introduction

Renal cell carcinomas (RCC) constitute 90–95% of the masses detected in the kidneys. RCCs are the third most common malignancy among urogenital tumors (1). Their prevalence has increased in Europe as well as globally in the last 20 years. The treatment comprises complete or partial surgical resection of the kidney (2). While all renal masses were treated with radical nephrectomy in the past, presently, partial nephrectomy has become the first option for treating renal masses. Initially, this technique was applied only in treating renal masses measuring less than 4 cm; however, nephron-sparing surgery (NSS) is used even in case of renal masses measuring more than 7 cm (3-5). According to the European Association of Urology (EAU) guidelines, NSS planning is suggested for treating all suitable T1a masses (6). Open, laparoscopic, and robotic application are the modes applied for partial nephrectomy. Many studies have demonstrated similar oncologic results for all three approaches. The main principle is to remove the mass altogether, leaving the maximal capacity of normal parenchymal tissues without any remaining tumoral tissues (7-10).

Methods such as wedge resection and enucleation are applied based on experience of the surgeon. After complete removal of mass, the opening is repaired primarily or by placing fat tissue or hemostatic agents, such as Surgicel and spongostan, to control bleeding (11).

Angiomyolipomas (AMLs) are considered as benign renal masses. In the past, these were considered hamartoma, but presently, these are formed by a heterogeneous tumor group. They generally contain fat, muscle, and vascular tissues (12). Although all AMLs are perivascular epithelioid cell tumors, most present different pathologies, imaging characteristics, and clinical behaviors. Since most AMLs contain a significant amount of fatty tissue, they are generally diagnosed using computed tomography (CT) or magnetic resonance imaging (MRI) by defining the imaging characteristics of fatty tissues in the mass (13). Although AMLs, which are high in fat and can be diagnosed through imaging, are called “classic AMLs,” recent developments have presented them as different types of renal AMLs. For example, it is known that, in addition to classic AMLs, some triphasic AMLs, known as low-fat AMLs, contain only a small amount of fatty cells and are sometimes mistaken for kidney cancers (14). The term “postsurgical fatty tumor” was not reported earlier in the literature. We defined this term as benign lesions that contain fatty tissues only and mimic AMLs in radiologic imaging, possibly because of repair with fatty tissue in the nephrectomy region in patients who underwent partial nephrectomy; however, these are not real AMLs.

Postsurgical fatty tumor is reported in imaging of some patients after NSS. Our aim in this study was to share information about the postsurgical fatty tumor that we detected after NSS but was not dealt with before in the literature. Further, we evaluated whether it was related to the applied surgical technique.

## Materials and Methods

Patients admitted to our urology polyclinic with different complaints or pre-diagnosis of renal mass between 2014 and 2021 were evaluated. They were applied open NSS following detection of renal mass. A total of 156 patients had partial nephrectomy. Nine patients with AMLs as primary pathology and four patients with surgical border positivity were excluded from the study. In all, 143 patients were included in the study.

After complete examination, findings such as complaints of patients at hospital admittance, presence of additional disease, preoperative mass dimension and localization, operative time, bleeding amount, duration of hospitalization, and pathology results were recorded. Contrasted abdomen CT (CACT) of all the included patients was taken preoperatively to evaluate renal mass. All patients also had thorax CT for preoperative staging.

RENAL nephrometry scoring system was used to decide surgical intervention. Pre- and postoperative hemogram and biochemistry measures were recorded and compared between two groups. All patients were divided into risk groups for routine control of 15 days after discharge. Postoperative follow-up protocol was evaluated as mentioned in EAU guidelines (15).

The follow-up protocol of the American Urological Association (AUA) and/or EAU guidelines was used for patient population (15, 16). Postoperative evaluation in low-risk patients was performed with ultrasonography (USG) in the 6th month, CACT in the 12th month, USG in the 2nd year, and CACT in the 3rd year. Average- and high-risk patients were evaluated with CACT in 6th, 12th, 24th, and 36th postoperative months. Patients with benign pathology were evaluated with USG in the 6th postoperative month. All USG and CACT findings of postoperative follow-ups of the patients were recorded.

The local human research ethics committee (Necmettin Erbakan University, Meram Medical Faculty Ethics Committee) approved the protocol “2015/236.” The analysis and data collection were performed following the Declaration of Helsinki after written informed consent was obtained from all patients.

### Surgical technique

Surgery was conducted under general anesthesia applied to all patients. Wedge resection or enucleation was planned based on preference of the surgeon or condition of the patient. The peritoneum was entered after anterior subcostal incision on the operation side. The descending or ascending colon was dissected based on positioning of tumor; the colon was freed through dissection, and the retroperitoneal area was reached after medialization. Gerota fascia and perirenal fatty tissue were completely dissected from the kidney and excised. Fatty tissue on the mass was excised and sent to pathology. Warm ischemia was performed on patients based on either tumor size and RENAL nephrometry scoring system or preference of the surgeon, or the surgery was completed without ischemia application. In ischemia-applied patients, surgical procedure was applied with the ischemia duration of less than 20 minutes. Tumor tissue was completely removed with 2–3-mm normal tissue from the border in patients applied wedge resection. In enucleation, the mass was completely removed by excising from pseudo capsule border through blunt dissection. The patients were divided into the following two groups based on the repair of tumor extraction region.

**Figure 1:**
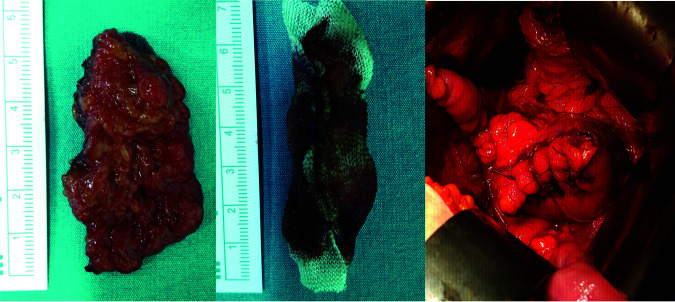
Perirenal adipose tissue taken from the non-tumoral area (left); appearance after the Surgicel is wrapped around the adipose tissue (middle); Surgicel-wrapped adipose tissue that was used for defect repair (right).

*Group 1*. Group in which fatty tissue was used for repair: It was prepared by wrapping as a roll with Surgicel around the fatty tissue taken from the non-tumoral area. Fatty tissue wrapped with hemostatic agent (SURGICEL^®^, Johnson & Johnson, Somerville, NJ), prepared in advance, was located in the opening and the tumor was removed from this area. The opening was closed with 2/0 absorbable suture. The technique applied is presented in Figure 1.

*Group 2*. Primary repair group: The opening where the tumor was removed was first closed with 3/0 absorbable suture and afterward with 2/0 absorbable suture. Surgicel and/or fibrin gel (FLOSEAL, Baxter Healthcare, Fremont, CA) was used as a hemostatic agent without using fatty tissue to stop bleeding between the sutures.

In either of the mentioned techniques, a catheter was placed in the perirenal area after control of bleeding, and the surgery ceased by suturing the layers in anatomic plane. Findings such as operative time and bleeding amount were recorded. Catheters placed in all the patients were removed on postoperative day 1, and spontaneous diuresis was observed. The patients were discharged after removal of catheters. They were called for routine control and follow-up in the 2nd week of discharge.

### Definition of Postsurgical Fatty Tumor

**Figure 2:**
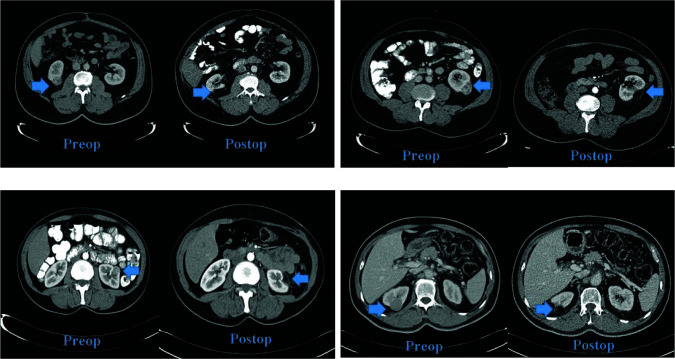
Image of postsurgical fatty tumor through contrast abdomen computed tomography.

Patients who did not have AMLs as primary pathology and had negative surgical border following partial nephrectomy were also defined for lesions detected radiologically and mimicking as AMLs. Such patients included fatty tissue only, did not present vascularization and contrasting, and had no dimensional increase in a minimum of 1-year follow-up. The radiologic image of postsurgical fatty tumor is presented in Figure 2.

### Statistical Analysis

Dataset analyses were made using the Statistical Package for Social Sciences volume 23.0 (IBM Corp., IL, Chicago). Continuous variables were presented as mean values and standard deviations. Independent *t*-test and Mann–Whitney U test were used for analyzing two groups. The Chi-square (χ^2^) test was applied for relationship analysis between categoric variables. P < 0.05 was accepted as statistically significant.

## Results

Partial nephrectomy was applied after retrospective data scanning in 143 patients. No difference was detected between the two groups regarding demographic and radiologic parameters such as the number of patients, age, gender, positioning of tumor, mass localization, tumor diameter, and RENAL nephrometry scoring system. Demographic and radiologic parameters of the groups are provided in Table 1.

**Table 1:** Demographic and radiologic parameters of the groups.

Variables		Group 1	Group 2	P-value
Number of patients		79	64	
Age (years)		57.3 (±11.4)	54.7 (±12.4)	0.186
Gender	F M	29 (36.7%) 50 (53.3%)	26 (40.6%) 38 (59.4%)	0.730
Side	Right Left	39 (49.4%) 40 (50.6%)	31 (48.4%) 33 (51.6%)	0.912
Localization	Lower pole Middle pole Upper pole	38 (48.1%) 23 (29.1%) 18 (22.8%)	31 (48.4%) 13 (20.3%) 20 (31.3%)	0.360
Tumor diameter (mm)		38.2 (±15.1)	41.1 (±16.9)	0.281
Renal score		5.2 (±1.3)	4.9 (±1)	0.107
Follow-up time (months)		34.6 (±17.4)	36.7 (±19.9)	0.104

Pathology of 119 patients (83.2%) in the entire patient population was detected as malign. The mean operative time was 101.3 ± 14.4 min (75–145 min), and the mean bleeding amount was measured as 263.9 ± 147.6 cc (50–750 cc). The median follow-up time was determined as 32 months (13–95 months). Evaluation of laboratory, preoperative, and postoperative parameter results of both groups demonstrated no significant difference other than the presence of postsurgical fatty tumor. Postsurgical fatty tumor in Group 1 was detected in 28 patients (35.4%), in which fat tissue was used for repair. In Group 2, the primary repair group, postsurgical fatty tumor was detected in just two patients (3.12%) (P < 0.001). In addition, a positive correlation was observed between the presence of postsurgical fatty tumor and the surgical technique used for fat tissue to repair (P < 0.001). Laboratory, preoperative, and postoperative results of both groups are provided in Table 2. It was observed that in the following period distant metastasis developed in four patients (2.8%). In addition, local relapse was observed in four patients (2.8%) during the follow-up period. There was no difference in metastasis and relapse rates between the both groups (P = 0.831 and P = 0.325).

**Table 2:** Laboratory, preoperative, and postoperative results of the groups.

Variables		Group 1	Group 2	P-value
Preop. hemoglobin (gm/dL)		14.3 (±1.7)	13.7 (±1.9)	0.051
Preop. creatinine (mg/dL)		0.88 (±0.23)	0.94 (±0.46)	0.145
Bleeding (mL)		254.2 (±140.9)	276.1 (±155.7)	0.379
Operation time (min)		99.7 (±15.2)	103.2 (±13.2)	0.146
Ischemia time (min)		14.8 (±2.3)	15.3 (±2)	0.074
Postop. hemoglobin (gm/dL)		12.6 (±1.9)	12.5 (±1.8)	0.75
Postop. creatinine (mg/dL)		0.91 (±0.25)	0.96 (±0.34)	0.124
Transfusion	No Yes	78 (98.7%) 1 (1.3%)	61 (95.3%) 3 (4.7%)	0.325
Complications				
Pathology	Benign malign	15 (19%) 64 (81%)	9 (14.1%) 55 (85.9%)	0.504
Oncocytoma Methanephric adenoma Hydatid cyst Pecoma Multicystic fibroma Renal cell carcinomas (RCC) Cystic RCC Malign mesenchymal tumor		7 (8.9%) 2 (2.5%) 3 (3.8%) 2 (2.5%) 1 (1.3%) 60 (75.9%) 4 (5.1%) 0	3 (4.7%) 1 (1.6%) 2 (3.1%) 2 (3.1%) 1 (1.6%) 53 (82.8%) 1 (1.6%) 1 (1.6%)	
Fuhrmann grade		1.8 (±1)	2 (±1)	0.130
Recurrence	No Yes	78 (98.7%) 1 (1.3%)	61 (95.3%) 3 (4.7%)	0.325
Metastasis	No Yes	77 (97.5%) 2 (2.5%)	62 (96.9%) 2 (3.1%)	0.831
Pseudoanjiomyolipoma	No Yes	51 (64.6%) 28 (35.4%)	62 (96.8%) 2 (3.12%)	<0.001

It was observed radiologically that the postsurgical fatty tumor in the nephrectomy region included fatty tissue only, and lacked vascularization and contrast involvement. No increase was observed in the mass size of detected postsurgical fatty tumor following a minimum of 1-year follow-up.

## Discussion

The use of NSS has risen by eight times in the last 20 years. It is used in 90% of T1a renal tumors (17). Enucleation and wedge resection are used as NSS methods. In both surgical methods different techniques and materials are used in renal defect area to cover collecting system and renal parenchyma and prevent postoperative bleeding following tumor resection (18). Urlesberger et al. stated that fibrin glues were used in renal parenchymal surgery (19). Levinson et al. reported providing successful hemostasis using fibrin glues in seven patients to repair following partial nephrectomy (20). Similarly, in a review, Ito et al. reported successful hemostasis with fibrin glues (21). Gill et al. compared Surgicel with the combination of gelatin matrix thrombin sealant (FLOSEAL, Baxter Healthcare, CA) for repair after laparoscopic partial nephrectomy. The authors stated that the use of combination was effective in lowering nonhemostatic complications(22) due to complete biocompatibility and feeding being performed through osmosis and not blood vessels (23). In the beginning, the kidney opening and closure technique is employed by locating the Surgicel-wrapped fatty tissue in the defective area of kidney parenchyma after tumor resection. Use of fatty tissue prevents urinary leakage, provides successful hemostasis, and a tension-free repair. Özkan et al. called this technique “lipocorticoplasty” in a study published in 2011 (24). We had applied repair with fatty tissue in 79 patients and fatty tissue-free repair in 64 patients. We observed no difference in oncologic results between the techniques. Similar bleeding amount and operation period were observed in both groups. During postoperative evaluation, we observed appearance of postsurgical fatty tumor in 28 (35.4%) patients in Group 1 using fatty tissue, and in just two patients (3.12%) in Group 2, primary repair group, not using fatty tissue for repair. These findings made us contemplate that AMLs reported during postoperative period were not actual AMLs but radiologically reported AMLs because of the fatty tissue used during surgery. We called them as postsurgical fatty tumor.

Angiomyolipomas are benign renal masses. Their prevalence changes approximately between 0.3% and –5% (25). They are defined as benign masses that generally have slow and uniform growth and cause minimal morbidity. Although 80% have a sporadic appearance and are unimportant, nearly 20% are related to tuberosclerosis. They can be detected randomly and may also cause clinical manifestations such as life-threatening bleeding (26). Histologically, renal AML can be classified as typical and atypical. Typical AMLs are triphasic and contain all three components in different proportions, namely, dilated blood vessels (angio), smooth muscle cells (myo), and mature adiposities (lipo). These three tissues are considered to develop from the same stem cell. Most AMLs belong to this group. However, some tumors consist of almost exclusively one component, while others are present in very small amounts. They are called monophasic AMLs, for example, the epitheloid variants of AMLs. Epitheloid AMLs contain no or very little fatty tissue. They generally contain numerous epithelioid muscle cells with abundant eosinophilic and granular cytoplasm. They may have aggressive local progress and present malign transformation. Histologically, they may be mistaken for RCC (27). Some authors claimed that renal AMLs could be classified radiologically through CT and MRI findings. They were classified as high-, low-, and no-fat AMLs based on the amount of fat detected in imaging studies (28). In literature, postsurgical fatty tumor was not reported in any definition.

We defined these lesions as postsurgical fatty tumor because they consisted of only fatty tissue and did not contain smooth muscle and vascular tissues. They are mistakenly reported as AMLs in postoperative period.

These findings make us believe that appearance of postsurgical fatty tumor is related to the fatty tissue located in the region because of the applied surgical technique. Although the employed surgical method has advantages, such as shorter operation duration, although statistically insignificant, misdiagnosis of AMLs during postoperative period is a primary disadvantage. However, we assumed that these lesions were not real AMLs but mistakenly reported as AMLs due to their highly fatty radiologic image. While most AMLs were followed up through active imaging, those causing clinical manifestations like pain or having bleeding risk must be treated. Treatment must also be planned in case of AMLs measuring more than 4–5 cm due to bleeding risk (12, 29). On the contrary, vascularization was not observed in the detected postsurgical fatty tumor. No increase was observed regarding their dimensions during follow-up. No additional treatment was required due to lack of vascularization, contrast involvement, or increase in dimensions.

Its higher prevalence in Group 1, using fatty tissue for repair, with appearance of postsurgical fatty tumor is a disadvantage of this technique. In the patients of Group 2 (primary repair group), in which fatty tissue was not used for repair, we believe the present image appeared due to other hemostatic agents used independently of fatty tissue.

Postsurgical fatty tumor appears as benign lesions not requiring additional treatment in evaluating masses detected postoperatively in patients who had partial nephrectomy and whose pathology was not reported as AMLs. We believe that postsurgical fatty tumor must be evaluated in distinctive diagnosis for evaluating these masses. Defined masses are observed in the partial nephrectomy region, free from postsurgical pathology. We believe that they are not actual AMLs, although they are generally diagnosed as AMLs in radiologic terms and appear like postsurgical fatty tumor as lipomatous tissue, probably because of the materials, such as fatty tissue, used during surgery.

## Conclusion

In patients with negative surgical margins after partial nephrectomy, lesions detected radiologically were defined as postsurgical fatty tumors. They contained only adipose tissue, and neither depicted vascularization and enhancement nor increase in size for at least 1 year. We believe that these lesions must be followed up as benign lesions, not requiring additional treatment.
